# Supplementary material for: Examination of a nutritional treatment pathway according to pretreatment health status and stress levels of patients undergoing hematopoietic stem cell transplantation
Source: PLoS One. 2022 Aug 1;17(8):e0271728. doi: 10.1371/journal.pone.0271728 (PMC9342724; doi:10.1371/journal.pone.0271728)
Supplement: Attachment — Submitted filename: Response to Reviewers.82608. docx (1).docx [file pone.0271728.s001.docx]

Dec15, 2021

Emily Chenette, Ph.D.

Editor-in-Chief

Senthilnathan Palaniyandi, Ph.D.

Academic Editor

PLOS ONE

Dear Dr. Chenette and Dr. Palaniyandi,

We wish to express our appreciation to the reviewers for their insightful comments on our manuscript, **“Examination of a nutritional treatment pathway according to pretreatment health status and stress levels of patients undergoing hematopoietic stem cell transplantation.”** Their comments helped us significantly improve the manuscript. Our specific responses to the reviewers’ comments are presented below, and revisions to the manuscript are indicated in yellow highlighted text. We hope that our responses and revisions to the manuscript are satisfactory. Thank you for the opportunity to submit our revised manuscript for consideration.

In addition, the following disclosures have been added:

Sources of financial support: This research project was sponsored by The Foundation for Promotion of Cancer Research and The Japan Dietetic Association under 2015 and 2016 Grants for Nutritional Guidance-related Research. The funders had no role in study design, data collection and analysis, decision to publish, or preparation of the manuscript.

Competing interests: The authors have declared that no competing interests exist.

Sincerely,

Takashi Aoyama, Ph.D.

Dietary Department

Shizuoka Cancer Center

1007 Shimonagakubo Nagaizumi-cho, Sunto-gun, Shizuoka 411-8777, Japan

Phone: +81-55-989-5222, Fax: 81-55-989-5634

E-mail: t.aoyama@scchr.jp

Reviewer comments

Reviewer #1: The authors' study underlines the importance of nutrition in allogeneic transplantation. This study is from Shizuoka in Japan. The patient numbers are low (40 patients treated with a myeloablative transplant). The authors claim that skeletal mass before transplant is an independent risk factor for death at 2 years, however they do not indicate what were the causes of death. This is important and should go into a multivariate analysis. Common causes of death after transplant are: infection, graft versus host disease, relapse of the malignancy. The literature is not up to-date. For example they fail to mention: Eglseer D et al recently published in BMT (341 patients), Tamaki M et al recently published in TCT (182 patients). The English needs improvements, for example in the legend of Fig 2 they indicate "3 cases of other cases" (??)

Reply: Thank you for your comment. We have revised the Results and Discussion sections and Figure 2 in order to include more background information.

Reviewer #2: The abstract section should be restructured into Introduction, Methods, Results, and Conclusion.

Reply: Thank you for your comment. We have revised the abstract to include this structured format.
